# National Outcomes of Cardiac Surgery in Patients with History of Bariatric Surgery

**DOI:** 10.1007/s11695-026-08548-x

**Published:** 2026-03-18

**Authors:** Andrew Tang, Jose Diz Ferre, Guangjin Zhou, Nicholas Schiltz, Edward Soltesz

**Affiliations:** 1 Department of Thoracic and Cardiovascular Surgery, Cleveland Clinic, Cleveland, United States; 2 Medical City Heart and Spine, Dallas, United States; 3 Department of Population and Quantitative Health Sciences, Case Western Reserve University, Cleveland, United States

**Keywords:** Cardiac surgery, Metabolic and Bariatric Surgery, Nationwide Inpatient Sample

## Abstract

**Background:**

The metabolic benefits of bariatric surgery are well established. However, its effects on cardiovascular surgery morbidity and mortality are not well understood.

**Methods:**

Between 2016 and 2021, the Nationwide Inpatient Sample included 343,975 discharges from bariatric surgery eligible candidates who underwent coronary artery bypass grafting, valve, aortic, or combination surgery. Among these were 11,035 (3.2%) patients with a diagnostic code for bariatric surgery. Balancing-score matching was used to mitigate confounding factors between patients who underwent bariatric surgery compared to their eligible counterparts who qualified for bariatric surgery but did not receive it. Obesity class subgroup analyses were also conducted following the same approach.

**Results:**

Among balancing-score–matched pairs, patients with bariatric surgery had lower hospital mortality (0.9% vs. 1.7% *P*=.03), fewer respiratory (pneumonia 4.2% vs. 6.9%, *P*<.001, respiratory failure 8.4% vs. 11%, *P*=.003) and infectious complications (1.2% vs. 2.0%, *P*=.03). However, in the obesity class 1 and 2 subgroups, outcomes were similar. Obesity class 3 patients with bariatric surgery had fewer pneumonia and infections compared to their counterparts.

**Conclusions:**

When comparing patients with and without a history of bariatric surgery, BMI may be an important confounder impacting cardiovascular surgery outcomes. Care teams should aim for metabolic optimization to improve surgical outcomes.

**Supplementary Information:**

The online version contains supplementary material available at 10.1007/s11695-026-08548-x.

## Key points

• Patients with bariatric surgery had lower in hospital mortality and morbidity.

• Hospital morbimortality was comparable among patients with obesity class 1 and 2.

• Care teams should prioritize metabolic optimization prior to cardiovascular surgery.

**Supplementary Information:**

The online version contains supplementary material available at 10.1007/s11695-026-08548-x.

## Introduction

In 2019, there was an estimated 160 million disability-adjusted life years related to obesity. [1] Disability-adjusted life years (DALY) is a measure of overall disease burden expressed as the number of years lost due to ill-health, disability, or early death. Obesity not only leads to cardiovascular diseases, but it also contributes to other cardiovascular risk factors such as dyslipidemia, type 2 diabetes, hypertension, and sleep disorders. [2] Thus far the most effective treatment for obesity has been for metabolic and bariatric surgery (MBS) with substantial durable weight loss. [2, 3].

Despite the well-established benefits of MBS, it is used as a treatment option in only 1.1% of eligible patients. [4]While in-hospital outcomes of patients with obesity have been studied after cardiovascular surgery [5], the association between MBS and cardiovascular outcomes remains unexplored in this population. Prior studies have found that MBS was associated with reduced mortality and respiratory complications. [6, 7] The purpose of this study was to analyze the association between MBS and cardiovascular surgery hospital morbidity and mortality. The secondary goal of this study was to delineate the effect of obesity class on cardiovascular surgery outcomes between patients who received MBS versus those who did not.

## Materials and Methods

### Data source and Study Design

The National Inpatient Sample (NIS) registry was queried for the years 2016–2021 to conduct this retrospective population-based cohort study. [8] This registry is part of the Healthcare Cost and Utilization Project’s database collection, which is the largest public inpatient data collection covering all-payers in the United States. The Institutional Review Board deemed this study exempt from review due to its use of publicly available, deidentified data.

## Study Population

Data on adult patients who underwent cardiovascular surgery were identified with International Classification of Diseases, 10th Version, Procedure Coding System (Table S1). Operations included coronary artery bypass grafting, valve, aortic or combination surgery. The study cohort consisted of patients with a history of MBS in the comparison group and eligible candidates for MBS but without a history of MBS as the control group. In accordance with the American Society for Metabolic and Bariatric Surgery, eligible candidates included patients with a body mass index (BMI) higher than 35, or 30 to 35 with concomitant metabolic conditions (Table S2). [4] Patients with nutritional deficiency, younger than 18 or older than 100 years, were excluded from the study. Patients with inaccurate, double, missing or non-obese BMI coding were grouped under BMI other and excluded from the subgroup analyses. There were 11,035 (3.2%) patients with a history of MBS and 332,940 (97%) candidates for MBS without it who met the criteria.

## Outcomes and Variables

Outcomes included hospital mortality and complications (Table S3). Patient-level as well as hospital-level variables were obtained from NIS data elements and comorbidities derived from the Elixhauser Comorbidity Index were considered. [9].

### Statistical Analysis

All analyses were conducted using the SAS System for Unix, version 9.4 (SAS Institute, Inc, Cary, NC). For descriptive and unadjusted analysis, we used NIS discharge survey weights to create national estimates. Unweighted counts and percentages were calculated after balancing-score match. The chi-square and Fisher’s exact test were used to compare categorical baseline characteristics.

Balancing scores were used to match covariables for patients with and without MBS to assess outcomes without influence from known confounders [10]. Covariables included procedure types, patient characteristics, hospital characteristics, insurance type, comorbidities, and region. To match those with and without MBS, a 1:1 greedy matching algorithm with a caliper of 0.2 times the standard deviation of the linear propensity score was performed.

Propensity matching resulted in 10,830 unweighted pairs of one patient with MBS and one eligible without MBS (Table S3). The balance of covariables before and after matching was also assessed by calculating the standardized differences between patients with and without MBS (Figure S1). [11] MBS eligible patients were further stratified into the 3 obesity class subgroups and adjusted for statistically significant differences within each subgroup. The purpose was to explore whether BMI acted as a confounding factor when analyzing the association of bariatric surgery and cardiovascular outcomes.

## Results

### Patient Characteristics

Patients with MBS were more likely to undergo valve surgery, and less likely to undergo CABG compared to obese patients without MBS (Table S4). Patients with MBS were more frequently be younger than 65 years of age, female, White, have private insurance and belonged to the highest income quartiles. Unsurprisingly, patients without MBS more often were BMI ≥ 35. Patients with MBS more often had comorbidities including autoimmune conditions, deficiency anemias, depression, hypothyroidism, neurological disease, psychoses, and peptic ulcer disease. However, obese patients without MBS had higher prevalences of complicated diabetes mellitus, fluid/electrolyte imbalance, and renal failure.

## Matched Hospital Outcomes

Among balancing-score–matched pairs, patients with MBS had lower in-hospital mortality (0.9% vs. 1.7% *P*=.03) (Table 1). Patients with MBS also had fewer complications (20% vs. 25%, *P* <.001), fewer pneumonia (4.2% vs. 6.9%, *P* <.001), less respiratory failure (8.4% vs. 11%, *P* =.003) and fewer infectious complications (1.2% vs. 2.0%, *P*=.03).

**Table 1:** Post balancing-score-matched hospital outcomes of patients with history of MBS vs. candidates without it

Variable	MBS *N* = 10,830	No MBS *N* = 10,830	*P* value
Hospital Mortality	100 (0.9%)	180 (1.7%)	0.03
Any complication	2,175 (20%)	2,705 (25%)	< 0.001
Stroke	415 (3.8%)	380 (3.5%)	0.56
Ischemic	410 (3.8%)	370 (3.4%)	0.5
Hemo	*	15 (0.1%)	0.32
Renal complication	90 (0.8%)	155 (1.4%)	0.06
Blood transfection due to bleed	130 (1.2%)	95 (0.9%)	0.32
Wound infection	25 (0.2%)	45 (0.4%)	0.28
Pneumonia	455 (4.2%)	750 (6.9%)	< 0.001
Respiratory failure	915 (8.4%)	1,205 (11%)	0.003
PE/DVT	85 (0.8%)	145 (1.3%)	0.08
GI Bleeding	260 (2.4%)	305 (1.8%)	0.4
Non-GI bleeding	290 (2.7%)	320 (3%)	0.59
Delirium	195 (1.8%)	245 (2.3%)	0.28
Infection or Sepsis	130 (1.2%)	220 (2%)	0.03

^*^Cells with frequency < 11 are altered to protect patient confidentiality and prevent the derivation of cells with frequency according to HCUP data use agreement

PE = pulmonary embolism; DVT = deep vein thrombosis

## Obesity Sub-Group Analysis

In the obesity subgroup analyses, the outcomes were comparable among patients with class 1 and 2 obesity (Tables 2 and 3). However, in patients with class 3 obesity, the ones with bariatric surgery had fewer pneumonia (2.6% vs. 7.6%, *P*<.001) and infections (1.2% vs. 4.5%, *P*=.003) (Table 4).

**Table 2:** Balancing-score matched obesity class 1 (BMI 30–35) subgroup analysis between patients with a history of MBS vs. eligible candidates (30–35 with a comorbidity) without it

Variable	MBS *N* = 1,665	No MBS *N* = 1,665	*P* value
Hospital Mortality	*	25 (1.5%)	0.10
Any complication	330 (20%)	335 (20%)	0.92
Stroke	75 (4.5%)	60 (3.6%)	0.55
Ischemic	70 (4.2%)	55 (3.3%)	0.54
Hemorrhagic	*	*	1.0
Renal complication	*	*	0.56
Blood transfusion	15 (0.9%)	15 (0.9%)	1.0
Wound infection	*	*	0.56
Pneumonia	70 (4.2%)	75 (4.5%)	0.85
Respiratory failure	125 (7.5%)	125 (7.5%)	1.0
PE/DVT	*	15 (0.9%)	0.32
GI Bleeding	*	*	1.0
Non-GI bleeding	30 (1.8%)	35 (2.1%)	0.77
Delirium	45 (2.7%)	30 (1.8%)	0.43
Infection or Sepsis	25 (1.5%)	*	0.26

^*^Cells with frequency < 11 are altered to protect patient confidentiality and prevent the derivation of cells with frequency according to HCUP data use agreement

PE = pulmonary embolism; DVT = deep vein thrombosis

**Table 3:** Balancing-score matched obesity class 2 (BMI 35–39) subgroup analysis between patients with a history of MBS VS. eligible candidates without it

Variable	MBS *N* = 1,790	No MBS *N* = 1,790	*P* value
Hospital Mortality	14 (0.8%)	25 (1.4%)	0.47
Any complication	355 (20%)	395 (22%)	0.47
Stroke	40 (2.2%)	70 (3.9%)	0.20
Ischemic	40 (2.2%)	70 (3.9%)	0.20
Hemorrhagic	0	0	N/A
Renal complication	*	*	1.0
Blood transfusion	*	20 (1.1%)	0.41
Wound infection	0	*	N/A
Pneumonia	75 (4.2%)	70 (3.9%)	0.85
Respiratory failure	170 (9.5%)	210 (12%)	0.34
PE/DVT	25 (1.4%)	30 (1.7%)	0.76
GI Bleeding	15 (0.8%)	*	0.32
Non-GI bleeding	25 (1.4%)	40 (2.2%)	0.40
Delirium	15 (0.8%)	35 (2%)	0.15
Infection or Sepsis	15 (0.8%)	15 (0.8%)	1.0

^*^Cells with frequency < 11 are altered to protect patient confidentiality and prevent the derivation of cells with frequency according to HCUP data use agreement

PE = pulmonary embolism; DVT = deep vein thrombosis

**Table 4:** Balancing-score matched obesity class 3 (BMI ≥ 40) subgroup analysis between patients with a history of MBS vs. eligible candidates without it

Variable	MBS *N* = 2,105	No MBS *N* = 2,105	*P* value
Hospital Mortality	15 (0.7%)	45 (2.1%)	0.07
Any complication	410 (20%)	585 (28%)	0.004
Stroke	70 (3.3%)	70 (3.3%)	1.0
Ischemic	70 (3.3%)	65 (3.1%)	0.84
Hemorrhagic	0	*	N/A
Renal complication	20 (1%)	50 (2.4%)	0.10
Blood transfusion	*	25 (1.2%)	0.25
Wound infection	*	15 (0.7%)	0.31
Pneumonia	55 (2.6%)	160 (7.6%)	< 0.001
Respiratory failure	235 (11%)	300 (14%)	0.16
PE/DVT	20 (1%)	30 (1.4%)	0.52
GI Bleeding	*	25 (1.2%)	0.10
Non-GI bleeding	40 (1.9%)	65 (3.1%)	0.26
Delirium	35 (1.7%)	20 (1%)	0.36
Infection or Sepsis	25 (1.2%)	95 (4.5%)	0.003

^*^Cells with frequency < 11 are altered to protect patient confidentiality and prevent the derivation of cells with frequency according to HCUP data use agreement

PE = pulmonary embolism; DVT = deep vein thrombosis

## Discussion

In terms of comorbidities, we observed that patients with MBS had a higher prevalence of autoimmune conditions, depression, hypothyroidism, and peptic ulcer disease that may have been detected during the MBS evaluation process. Regarding deficiency anemia, this could have been a consequence of the MBS as this is a common morbidity of this procedure [12], as well as the lower prevalence of complicated diabetes mellitus.

Regarding the findings of this study, patients with a history of MBS had significantly lower in-hospital mortality, fewer respiratory and infectious complications compared to MBS-eligible counterparts. While these differences were not observed in class 1 and 2 groups, patients with obesity class 3 and a history of MBS had fewer pneumonia and infections compared to their non-MBS counterparts. Due to the multisystem benefits of MBS, there may be several mechanisms as to why patients with a history of MBS perform better after cardiovascular surgery compared to their obese counterparts. Metabolic and bariatric surgery induces durable improvements in insulin sensitivity [13], and systemic inflammation [14], which can affect postoperative outcomes [15]. Metabolic and bariatric surgery is also associated with improved LV geometry [16], and lower pulmonary artery pressures [17], which may translate to improved hemodynamic stability in the postoperative setting. Additionally, there is a well-established restrictive effect on pulmonary function from obesity. This can create difficulty performing good bronchopulmonary hygiene in the postoperative setting, which increases the likelihood of pneumonia and respiratory compromise.

Interestingly, these benefits disappeared in the obesity class subgroup analysis. Some benefits in the MBS group, such as the decrease in pneumonia and infections, remain only among patients with obesity class 3. This highlights the role of BMI as a confounding factor in the association between the history of bariatric surgery and cardiovascular hospital outcomes. The lower prevalence of morbidity and mortality in lower BMI ranges also suggests the lack of obesity paradox previously observed. [5, 18–20].

In terms of clinical practice, it may not always be feasible to undergo MBS prior to cardiovascular surgery. Institutions may consider prehabilitation—including medically supervised weight reduction, exercise therapy, and nutritional support—prior to elective cardiovascular surgery. Prehabilitation has been associated with reduced postoperative complications [21]. Where MBS is not feasible, medical weight loss with pharmacologic agents such as GLP-1 receptor agonists may offer preoperative improvements [22].

### Limitations

The NIS is an administrative database that is constructed around billing information; therefore, it only provides discharge-level information. This study is also subject to selection bias given its retrospective cohort design. Despite using balancing-score matching to control baseline differences in patient cohorts, we could not exclude residual or unmeasured confounding due to the observational nature of this study. Furthermore, there is a lack of granular data regarding the exact type of MBS, time elapsed since MBS, degree of postoperative weight loss, persistent vs. recurrence of obesity, metabolic response (remission of diabetes, hypertension, NAFLD, OSA, etc.) and whether BMI at the time of cardiac surgery reflects a true post-MBS physiology. Unfortunately, physiology after MBS varies widely, and postoperative outcomes after cardiac surgery depend heavily on the patient’s metabolic status at the time of cardiovascular surgery.

## Conclusions

Care teams should prioritize metabolic optimization for improved hospitality mortality, respiratory and infectious complications. Although MBS appears to have a protective effect, this may be mediated by BMI. When stratifying by obesity class, MBS was associated with improved respiratory and infectious outcomes compared to their non-MBS counterparts only among patients with obesity class 3.

## Supplementary Information

Below is the link to the electronic supplementary material.

Supplementary Material 1

Supplementary Material 2

## Data Availability

The data that support the findings of this study are publicly available.
