# Supplementary material for: National Outcomes of Cardiac Surgery in Patients with History of Bariatric Surgery
Source: Obes Surg. 2026 Mar 18;36(4):1623–7. doi: 10.1007/s11695-026-08548-x (PMC13083515; doi:10.1007/s11695-026-08548-x)
Supplement: Supplementary file 1 — Supplementary Material 1 [file 11695_2026_8548_MOESM1_ESM.docx]

Supplemental Information

**Table S1.** ICD-10-PCS Codes of Inclusion Criteria for the Cohort

| **Variable** | **ICD10 codes** |
| --- | --- |
| Coronary artery bypass grafting | 02100, 02110, 02120, 02130 |
| Aortic valve replacement or repair | 02QF0ZZ, 02RF07Z, 02RF08Z, 02RF0JZ, 02RF0KZ |
| Mitral valve replacement or repair | 02QG0ZE, 02QG0ZZ, 02RG07Z, 02RG08Z, 02RG0JZ, 02RG0KZ |
| Tricuspid valve replacement or repair | 02QJ0ZG, 02QJ0ZZ, 02RJ07Z, 02RJ08Z, 02RJ0JZ, 02RJ0KZ |
| Pulmonic valve replacement or repair | 02QH0ZZ, 02RH07Z, 02RH08Z, 02RH0JZ, 02RH0KZ |
| Aortic procedures | 02BW0ZZ, 02BX0ZZ, 02QX0ZZ, 02QW0ZZ, 02RX07Z, 02RX08Z, 02RX0JZ, 02RX0KZ, 02RW07Z, 02RW08Z, 02RW0JZ, 02RW0KZ |

**Table S2.** ICD-10-CM Codes Used to Identify History of Metabolic and Bariatric Surgery and Eligible Patients

| Z98.84, Z68.30- Z68.45, E11, I10, E88.81, G47.33, I25.1, I50.22, I48, J45, K76.0, K75.81, N18, E28.2, N46.1, N97.0, K21, G93.2, M25.9: diabetes type 2, primary hypertension, metabolic syndrome, obstructive sleep apnea, atherosclerotic heart disease of native coronary artery, chronic systolic (congestive) heart failure, atrial fibrillation and flutter, asthma, fatty (change of) liver not elsewhere classified nonalcoholic fatty liver disease, nonalcoholic steatohepatitis, chronic kidney disease, polycystic ovarian syndrome, male infertility due to oligospermia, female infertility associated with anovulation, gastro-esophageal reflux disease, benign intracranial hypertension, joint disorder, unspecified |
| --- |

**Table S3. ICD-10-CM Code Description for complications**

| Complication | ICD10 codes |
| --- | --- |
| Ischemic Stroke | G43601 G43609 G43611 G43619 I6300 I63011 I63012 I63013 I63019 I6302 I63031 I63032 I63033 I63039 I6309 I6310 I63111 I63112 I63113 I63119 I6312 I63131 I63132 I63133 I63139 I6319 I6320 I63211 I63212 I63213 I63219 I6322 I63231 I63232 I63233 I63239 I6329 I6330 I63311 I63312 I63313 I63319 I63321 I63322 I63323 I63329 I63331 I63332 I63333 I63339 I63341 I63342 I63343 I63349 I6339 I6340 I63411 I63412 I63413 I63419 I63421 I63422 I63423 I63429 I63431 I63432 I63433 I63439 I63441 I63442 I63443 I63449 I6349 I6350 I63511 I63512 I63513 I63519 I63521 I63522 I63523 I63529 I63531 I63532 I63533 I63539 I63541 I63542 I63543 I63549 I6359 I636 I638 I6381 I6389 I639 I6930 I6931 I69310 I69311 I69312 I69313 I69314 I69315 I69318 I69319 I69320 I69321 I69322 I69323 I69328 I69331 I69332 I69333 I69334 I69339 I69341 I69342 I69343 I69344 I69349 I69351 I69352 I69353 I69354 I69359 I69361 I69362 I69363 I69364 I69365 I69369 I69390 I69391 I69392 I69393 I69398 I6980 I6981 I69810 I69811 I69812 I69813 I69814 I69815 I69818 I69819 I69820 I69821 I69822 I69823 I69828 I69831 I69832 I69833 I69834 I69839 I69841 I69842 I69843 I69844 I69849 I69851 I69852 I69853 I69854 I69859 I69861 I69862 I69863 I69864 I69865 I69869 I69890 I69891 I69892 I69893 I69898 I6990 I6991 I69910 I69911 I69912 I69913 I69914 I69915 I69918 I69919 I69920 I69921 I69922 I69923 I69928 I69931 I69932 I69933 I69934 I69939 I69941 I69942 I69943 I69944 I69949 I69951 I69952 I69953 I69954 I69959 I69961 I69962 I69963 I69964 I69965 I69969 I69990 I69991 I69992 I69993 I69998 |
| Hemorrhagic Stroke | I6000 I6001 I6002 I6010 I6011 I6012 I6020 I6021 I6022 I6030 I6031 I6032 I6050 I6051 I6052 I602 I604 I606 I607 I608 I609 I610 I611 I612 I613 I614 I615 I616 I618 I619 I621 I629 I6200 I6201 I6202 I6203 I6900 I6901 I6920 I6921 I6901 I6910 I6911 I69010 I69011 I69012 I69013 I69014 I69015 I69018 I69019 I69020 I69021 I69022 I69023 I69028 I69031 I69032 I69033 I69034 I69039 I69041 I69042 I69043 I69044 I69049 I69051 I69052 I69053 I69054 I69059 I69061 I69062 I69063 I69064 I69065 I69069 I69090 I69091 I69092 I69093 I69098 I69110 I69111 I69112 I69113 I69114 I69115 I69118 I69119 I69120 I69121 I69122 I69123 I69128 I69131 I69132 I69133 I69134 I69139 I69141 I69142 I69143 I69144 I69149 I69151 I69152 I69153 I69154 I69159 I69161 I69162 I69163 I69164 I69165 I69169 I69190 I69191 I69192 I69193 I69198 I6920 I69210 I69211 I69212 I69213 I69214 I69215 I69218 I69219 I69220 I69221 I69222 I69223 I69228 I69231 I69232 I69233 I69234 I69239 I69241 I69242 I69243 I69244 I69249 I69251 I69252 I69253 I69254 I69259 I69261 I69262 I69263 I69264 I69265 I69269 I69290 I69291 I69292 I69293 I69298 |
| Renal Complication | N170 N171 N172 N178 N179 N990 N19 AND 5A1D00Z 5A1D60Z 5A1D70Z 5A1D80Z 5A1D90Z EXCLUDING Z992 |
| Pneumonia | A0103 A0222 A202 A212 A221 A310 A3791 A430 A481 B012 B052 B0681 B250 B371 B380 B381 B382 B390 B391 B392 B583 B59 B7781 J120 J121 J122 J123 J1281 J1289 J129 J13 J14 J150 J151 J1520 J15211 J15212 J1529 J153 J154 J155 J156 J157 J158 J159 J160 J168 J180 J181 J188 J189 J690 J691 J698 J851 J930 J9311 J9381 J9382 J9383 J939 |
| Respiratory Failure | J951 J952 J953 J954 J955 J9500 J9501 J9502 J9503 J9504 J9509 J9561 J9562 J9571 J9572 J9588 J9589 J9584 J95811 J95812 J95821 J95822 J95830 J95831 J95850 J95851 J95859 J95860 J95861 J95862 J95863 |
| Pulmonary embolism and deep venous thrombosis | I2602 I2609 I2692 I2693 I2694 I2699 I824 I8262 |
| Gastrointestinal bleeding | K921 K922 K31811 I8501 I8511 K2211 K2971 K2901 K2961 K2991 K250 K252 |
| Non-gastrointestinal bleeding | I974 I976 T82837 D683 D699 I230 I312 S26.0 K66.1 R58 |
| Delirium | F05 |
| Infection or Sepsis | T8140 T8141 T8142 T8143 T8149 A49 T8144 A419 R6520 R6521 |

**Supplemental Figure 1.** Standardized Difference Plot before and after balance-score matching.


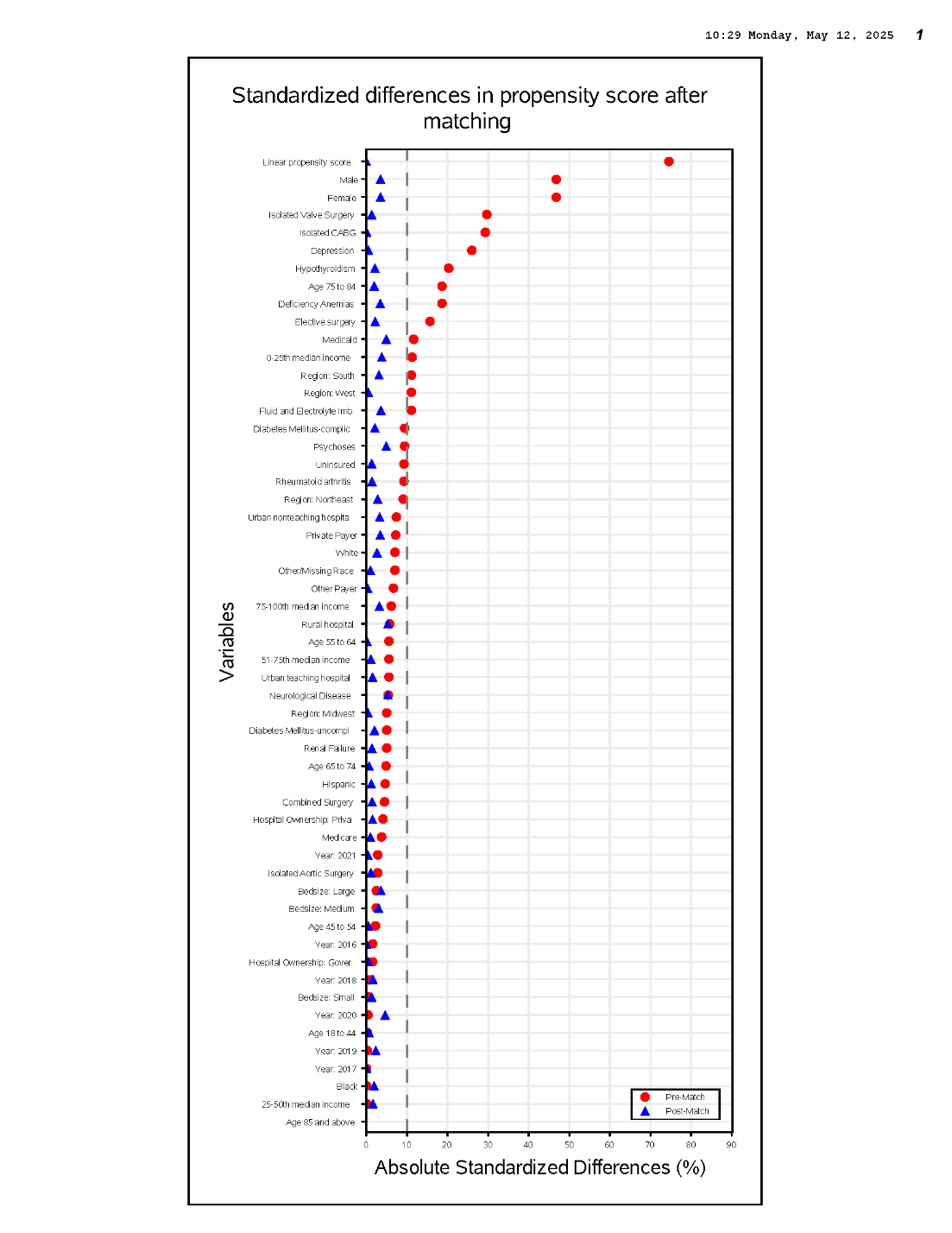


**Table S4.** Pre- Balancing-Score Match Characteristics of Patients Undergoing Cardiovascular Surgery According to History of MBS.

| **Variable** | **MBS**  **N = 11,035** | | **No MBS**  **N = 332,940** | | ***P* value** |
| --- | --- | --- | --- | --- | --- |
| **Surgery types** |  |  |  |  |  |
| CABG only | 6,285 | 57% | 235,975 | 71% | <.001 |
| Valve only | 3,030 | 28% | 51,405 | 15% | <.001 |
| Aorta only | 300 | 2.7% | 7,595 | 2.3% | .17 |
| Combination | 1,420 | 13% | 37,965 | 11% | .03 |
| **Age category** |  |  |  |  | <.001 |
| 18-44 | 530 | 4.8% | 15,745 | 4.7% |  |
| 45-54 | 1,670 | 15% | 47,755 | 14% |  |
| 55-64 | 3,790 | 34% | 105,510 | 32% |  |
| 65-74 | 4,280 | 39% | 121,175 | 36% |  |
| 75-84 | 760 | 6.9% | 41,235 | 12% |  |
| 85+ | * | * | 1,520 | 0.5% |  |
| **BMI** |  |  |  |  | N/A |
| BMI>35 | 3,920 | 36% | 193,960 | 58% |  |
| BMI 30-34.9 | 1,665 | 15% | 138,980 | 42% |  |
| Other | 5,450 | 49% | 0 | 0.0% |  |
| **Sex** |  |  |  |  | <.001 |
| Male | 5,050 | 46% | 227,430 | 68% |  |
| Female | 5,985 | 54% | 105,480 | 32% |  |
| Missing | 0 | 0.0% | 30 | 0.0% |  |
| **Race** |  |  |  |  | .002 |
| White | 8,885 | 80% | 258,400 | 78% |  |
| Black | 860 | 7.8% | 25,900 | 7.8% |  |
| Hispanic | 640 | 5.8% | 23,095 | 6.9% |  |
| Other** | 650 | 5.9% | 25,545 | 7.7% |  |
| **Admission status** |  |  |  |  | <.001 |
| Elective | 4,260 | 39% | 153,835 | 46% |  |
| Urgent | 6,755 | 61% | 178,205 | 54% |  |
| Missing | 20 | 0.2% | 900 | 0.3% |  |
| **Median Household Income** |  |  |  |  | <.001 |
| 1st quartile (lowest) | 2,465 | 22% | 90,580 | 27% |  |
| 2nd quartile | 3,120 | 28% | 94,205 | 28% |  |
| 3rd quartile | 2,995 | 27% | 82,260 | 25% |  |
| 4th quartile (highest) | 2,290 | 21% | 61,005 | 18% |  |
| Missing | 165 | 1.5% | 4,890 | 1.5% |  |
| **Insurance** |  |  |  |  | <.001 |
| Medicaid | 605 | 5.5% | 28,110 | 8.4% |  |
| Medicare | 5,625 | 51% | 163,630 | 49% |  |
| Other | 250 | 2.3% | 11,190 | 3.4% |  |
| Private | 4,395 | 40% | 120,845 | 36% |  |
| Uninsured | 145 | 1.3% | 8,685 | 2.6% |  |
| Missing | 15 | 0.1% | 480 | 0.1% |  |
| **Teaching status of hospital** |  |  |  |  | .002 |
| Metropolitan non-teaching | 215 | 1.9% | 9,355 | 2.8% |  |
| Metropolitan teaching | 1,270 | 12% | 44,310 | 13% |  |
| Non-metropolitan hospital | 9,550 | 86% | 279,275 | 84% |  |
| **Hospital Region** |  |  |  |  | <.001 |
| Northeast | 2,180 | 20% | 54,180 | 16% |  |
| Midwest | 2,860 | 26% | 93,870 | 28% |  |
| South | 3,870 | 35% | 134,710 | 40% |  |
| West | 2,125 | 19% | 50,180 | 15% |  |
| **Hospital bed size** |  |  |  |  | .53 |
| Small | 1,225 | 11% | 37,600 | 11% |  |
| Medium | 2,575 | 23% | 81,065 | 24% |  |
| Large | 7,235 | 66% | 214,275 | 64% |  |
| **Calendar year** |  |  |  |  | .9 |
| 2016 | 1,705 | 16% | 53,300 | 16% |  |
| 2017 | 1,840 | 17% | 55,430 | 17% |  |
| 2018 | 1,890 | 17% | 58,000 | 17% |  |
| 2019 | 1,900 | 17% | 57,595 | 17% |  |
| 2020 | 1,680 | 15% | 51,170 | 15% |  |
| 2021 | 2,020 | 18% | 57,445 | 17% |  |
| **Hospital Ownership** |  |  |  |  | .17 |
| Government | 815 | 7.4% | 25,950 | 7.8% |  |
| Private | 9,015 | 82% | 266,470 | 80% |  |
| Private, investor-owned | 1,205 | 11% | 40,520 | 12% |  |
| **Comorbidities** |  |  |  |  |  |
| AIDS | * | * | 245 | 0.1% | .63 |
| Alcohol abuse | 260 | 2.4% | 8,890 | 2.7% | .36 |
| Autoimmune conditions | 435 | 3.9% | 7,720 | 2.3% | <.001 |
| Arrythmia | 5,295 | 48% | 159,280 | 48% | .89 |
| Deficiency anemias | 2,360 | 21% | 47,545 | 14% | <.001 |
| Chronic blood loss anemia | 110 | 1.0% | 3,200 | 1.0% | .86 |
| Congestive heart failure | 110 | 1.0% | 4,670 | 1.4% | .11 |
| Chronic lung disease | 2,420 | 22% | 77,055 | 23% | .18 |
| Coagulopathy | 2,865 | 26% | 81,500 | 24% | .11 |
| Depression | 2,230 | 20% | 36,235 | 11% | <.001 |
| Diabetes mellitus - uncomplicated | 2,155 | 20% | 58,395 | 18% | .02 |
| Diabetes mellitus-complicated | 3,755 | 34% | 128,420 | 39% | <.001 |
| Drug abuse | 220 | 2.0% | 5,375 | 1.6% | .17 |
| Fluid and electrolyte imbalance | 3,840 | 35% | 133,700 | 40% | <.001 |
| Liver disease | 335 | 3.0% | 11,405 | 3.4% | .3 |
| Hypertension | 6,860 | 62% | 203,065 | 61% | .28 |
| Hypothyroidism | 2,235 | 20% | 42,420 | 13% | <.001 |
| Lymphoma | 40 | 0.4% | 1,040 | 0.3% | .68 |
| Metastatic cancer | 15 | 0.1% | 565 | 0.2% | .7 |
| Neurological disease | 740 | 6.7% | 18,135 | 5.4% | .01 |
| Obesity | 6,540 | 59% | 332,935 | 100% | <.001 |
| Paralysis | 270 | 2.4% | 7,635 | 2.3% | .64 |
| Peripheral vascular disease | 1,565 | 14% | 45,430 | 14% | .49 |
| Psychoses | 375 | 3.4% | 6,315 | 1.9% | <.001 |
| Pulmonary circulation disorders | * | 0.1% | 425 | 0.1% | .63 |
| Renal failure | 1,705 | 16% | 57,605 | 17% | .02 |
| Solid tumor without metastasis | 110 | 1.0% | 3,140 | 0.9% | .8 |
| Peptic ulcer disease | 125 | 1.1% | 1,865 | 0.6% | <.001 |
| Valvular disease | 90 | 0.8% | 2,535 | 0.8% | .77 |
| Weight loss | 260 | 2.4% | 9,785 | 2.9% | .12 |

Individuals may have more than one comorbidity; therefore, comorbidity sub-categories are not mutually exclusive.

AIDS = acquired immunodeficiency syndrome; CABG = coronary artery bypass surgery; yrs = years

^*^Cells with frequency <11 are altered to protect patient confidentiality and prevent the derivation of cells with frequency according to HCUP data use agreement

^a^Other race includes Native American, Asian or Pacific Islander, other race/mixed race
